# Chromosome Diversity and Evolution of the Endemic Malagasy Velvet Geckos of the Genus *Blaesodactylus* (Reptilia, Gekkonidae)

**DOI:** 10.3390/ani13132068

**Published:** 2023-06-22

**Authors:** Marcello Mezzasalma, Elvira Brunelli, Gaetano Odierna, Fabio Maria Guarino

**Affiliations:** 1 Department of Biology, Ecology and Earth Science, University of Calabria, Via P. Bucci 4/B, 87036 Rende, Italy; 2 Department of Biology, University of Naples Federico II, Via Cinthia 26, 80126 Naples, Italy; gaetanodierna@gmail.com (G.O.); fabio.guarino@unina.it (F.M.G.)

**Keywords:** cytogenetics, evolution, karyotype, Madagascar, Squamata

## Abstract

**Simple Summary:**

We implemented a molecular and phylogenetic analysis and a comparative karyological investigation with standard and chromosome banding methods on different taxa of the endemic Malagasy velvet geckos of the genus *Blaesodactylus*. We present the description of the karyotype of three different taxa and a characterization of the chromosomal diversity in the genus. We show the occurrence of karyological variability in the genus *Blaesodactylus* in terms of chromosome number (2n = 40–42), morphology, chromosome position of loci of NORs, and distribution pattern of heterochromatin. Considering our results together with the available information on evolutionary related gecko species, we hypothesize that the karyotype evolution in *Blaesodactylus* mostly involved a decrease in the total number of chromosomes and the formation of biarmed elements. We also highlight that similar pathways of chromosomal rearrangements have been previously observed in other geckos, possibly representing a convergent karyotype evolution.

**Abstract:**

We performed a molecular and phylogenetic analysis and a comparative cytogenetic study with standard karyotyping, silver staining (Ag-NOR) and sequential C-banding + Giemsa, + fluorochromes on several *Blaesodactylus* samples. The phylogenetic inference retrieved two main clades, the first comprises *B. victori*, *B. microtuberculatus* and *B. boivini*, while the second includes *B. sakalava*, *B. antongilensis* and *B. ambonihazo*. The available samples of *B. sakalava* form two different clades (here named *B. sakalava* clade A and clade B), which probably deserve a taxonomic re-evaluation. We found a karyological variability in *Blaesodactylus* in terms of chromosome number (2n = 40–42), morphology, location of NORs, and heterochromatin distribution pattern. *Blaesodactylus antongilensis* and *B. sakalava* clade A and B showed a karyotype of 2n = 40 mostly telocentric chromosomes. Pairs 1 and 6 were metacentric in *B. sakalava* clade A and B, while pair 1 was composed of subtelocentric/submetacentric elements in *B. antongilensis.* In contrast, *B. boivini* displayed a karyotype with 2n = 42 only telocentric chromosomes. NORs were on the first chromosome pair in *B. boivini*, and on the second pair in *B. antongilensis*. Adding our data to those available from the literature on evolutionarily related species, we highlight that the chromosome diversification in the genus probably proceeded towards a progressive reduction in the chromosome number and the formation of metacentric elements.

## 1. Introduction

Madagascar is well-known for its extraordinary biodiversity and remarkable degree of endemism and represents a unique model region for evolutionary studies [1,2,3]. The terrestrial reptile fauna of the island includes more than 450 endemic species of squamates belonging to six families of snakes (Boidae, Elapidae, Psammophiidae, Pseudoxyrhophiidae, Typhlopidae, Xenotyphlopidae) and six families of lizard (Agamidae, Chamaeleonidae, Gekkonidae, Gerrhosauridae, Opluridae, and Scincidae) [4,5]. Among them, the Malagasy Gekkonidae include eleven different genera (*Blaesodactylus*, *Ebenavia*, *Geckolepis*, *Gehyra*, *Hemidactylus*, *Lygodactylus*, *Matoatoa*, *Paragehyra*, *Paroedura*, *Phelsuma* and *Uroplatus*) and more than 140 currently described species [5]. Nevertheless, although there has been significant progress achieved in the last few decades, the diversity of Malagasy reptiles is still relatively poorly known, with several new species discovered every year [4,5]. Even if recent research began to better describe the taxonomy and the evolutionary relationships of several different groups, only a small fraction of Malagasy reptile species has been analyzed with cytogenetic methods, despite increasing evidence that their diversity is reflected at the karyotypic level (see e.g., [6,7,8,9]). In fact, karyotype changes may either precede or follow molecular evolution, directly promoting cladogenesis or deriving from phylogenetic divergence [10,11]. In either circumstance, different karyological characteristics (e.g., different ploidy, total haploid number of chromosomes, chromosome morphology, presence or absence of differentiated sex chromosomes and occurrence and localization of particular DNA sequences) represent discrete cytogenetic markers which are helpful to describe different evolutionary trends or apomorphisms (see e.g., [12,13,14]).

Furthermore, squamates reptiles represent emergent model organisms in evolutionary cytogenetics as they are characterized by a high variability in chromosome number (from 2n = 16 to 2n = 62) and morphology and by the evolution of simple (XY, ZW) and multiple sex chromosome systems (X_1_X_2_Y and Z_1_Z_2_W) with either male or female heterogamety (see e.g., [15]). Two different general karyotype organizations have been described in squamates as “asymmetrical” (with macro- and microchromosomes) and “symmetrical” (with chromosomes that gradually decrease in length). Asymmetrical karyotypes are common in Iguania, many Scincomorpha and Platynota, while symmetrical karyotypes are often found in Lacertidae and Gekkota (see e.g., [15]).

The genus *Blaesodactylus* currently includes six described species (*B. ambonihazo*, *B. antongilensis*, *B. boivini*, *B. victori*, *B. microtuberculatus* and *B. sakalava*), which have been recently studied with morphological and molecular methods [16,17,18,19]. In contrast, chromosome analyses have been performed so far only on samples ascribed to *B*. *boivini* based on morphological characters [20], leaving the karyological variability of the genus completely unexplored. Concerning *B*. *boivini*, Chrostek et al. [20] analyzed one male and one female sample of the species and described a karyotype composed of 2n = 42, with only telocentric chromosomes which gradually decrease in length. Blocks of heterochromatin were identified on the centromeres of the six largest chromosome pairs and both sexes showed the same chromosome complement, without any evident heteromorphic sex chromosome pair. NORs were identified on the centromeric region of the first chromosome pair and telomeric repeats were localized at the centromeres of all chromosome pairs as well as at interstitial positions of several pairs [20].

In this paper we performed a preliminary molecular and phylogenetic analysis, using a segment of the mitochondrial ND4, and a comparative cytogenetic study with standard karyotyping, Ag-NOR staining and sequential C-banding on different *Blaesodactylus* samples from distinct Malagasy areas. We provide the first karyotype description of different taxa of the genus and a characterization of their chromosomal diversity. We show that chromosome variability in terms of total chromosome number, number of uni- and biarmed chromosomes and a different localization of chromosomal markers (NORs loci and heterochromatic regions) characterizes the studied taxa of the genus *Blaesodactylus*.

Finally, adding our newly generated karyotype data to those already available from the literature on evolutionarily closely related species and genera, we advance a hypothesis on the chromosome diversification in different genera of the family Gekkonidae.

## 2. Material and Methods

### 2.1. Sampling

We studied ten specimens of three species of Malagasy geckos of the genus *Blaesodactylus*. The taxonomic attribution after the molecular analysis (see below), field number, sex, and origin of all the samples analyzed in this study are provided in Table 1. All the specimens used in the molecular and cytogenetic analyses (Table 1) were collected between 1997–2002 for other research purposes and no animal was sampled during the realization of this study. After capture, animals were injected with a 0.5 mg/mL colchicine solution (0.1 mL/10 g body weight). Tissue samples (intestine, spleen and gonads) were incubated for 30 min in hypotonic solution (KCl 0.075 M + sodium citrate 0.5%, 1:1), fixed and conserved in Carnoy’s buffer solution (methanol and acetic acid, 3:1). The fixed material was then preserved at 4 °C and transferred to the laboratory of University of Naples Federico II where it was processed as described below.

### 2.2. Molecular and Phylogenetic Analysis

A molecular and phylogenetic analysis was performed to ascertain the taxonomic status and the phylogenetic position of all the samples studied and to associate DNA sequences to the newly described karyotypes. The molecular analysis was performed using a fragment of the mitochondrial NADH dehydrogenase subunit 4 (ND4), which has been previously used in phylogenetic inferences in the genus *Blaesodactylus* [16,17,19,21]. Total genomic DNA was extracted following Sambrook et al. [22] and PCR amplification of the chosen ND4 fragment was performed according to Bauer et al. [17]. Amplicons were sequenced on an automated sequencer ABI 377 (Applied Biosystems, Foster City, CA, USA) using BigDye Terminator 3.1 (ABI). Chromatograms were checked and manually edited using Chromas Lite 2.6.6 and BioEdit 7.2.6.1 [23] and compared with available homologous traits deposited in GenBank. All the newly determined sequences were deposited in GenBank (accession numbers: OR113357-OR113363). A phylogenetic analysis using the newly determined ND4 sequences along with the homologous traits presented by Ineich et al. [19] (accession numbers: KX101035-KX101047, including those of the samples FGMV 2029, FGMV 2030 and FGMV 2032, here used in the cytogenetic analysis) was performed using MrBayes 3.2 [24]. The best fitting substitution model was selected using jModeltest 2.1.7 under the corrected Akaike information criterion (AICc) [25]. We run two independent Monte Carlo Markov Chains (MCMC) for 8,000,000 generations, sampling the chains every 1000 generations and discarding the first 25% of the trees sampled as burn-in. We used as the outgroup the homologous ND4 trait of *Geckolepis maculata* (accession number JQ974269).

### 2.3. Cytogenetic Analysis

Chromosomes were obtained from preserved tissue samples and cell suspensions using the air-drying method, as described in Mezzasalma et al. [9]. The chromosome analysis was performed with standard karyotyping (5% Giemsa solution at pH 7 for 10 min) and different chromosome staining and banding methods. C-banding was performed following Sumner [26], and sequential C-banding + CMA_3_ + DAPI according to Mezzasalma et al. [27]. Active nucleolus organizing regions (NORs) were identified following the Ag-NOR staining method described by Howell and Black [28]. Metaphase plates were scored and recorded with an optical and an epifluorescence microscope (Axioscope Zeiss) equipped with an image analysis system. Karyotype reconstruction and calculation of chromosome relative length (RL = length of a chromosome/total karyotype length) (see Table 2) were performed after scoring at least ten metaphase plates per sample. Chromosomes were classified following Levan et al. [29] in metacentric (m), submetacentric (sm), subtelocentric (st) and telocentric (t).

## 3. Results

### 3.1. Molecular and Phylogenetic Analysis

**Figure 1:**
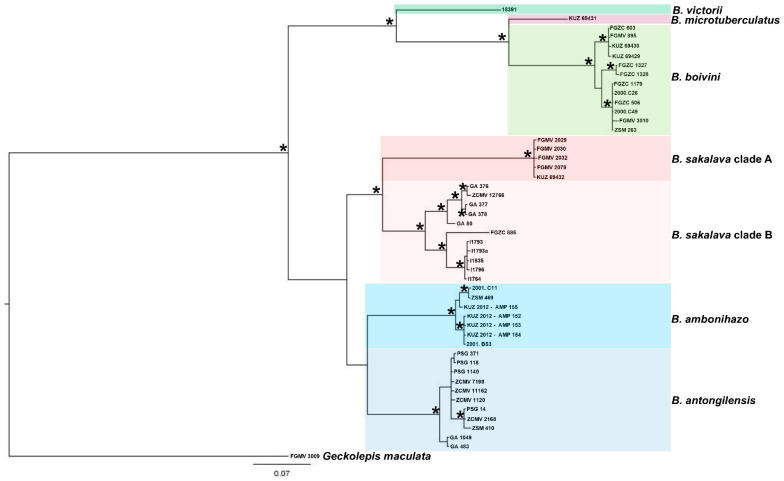
Phylogenetic tree with Bayesian inference using MrBayes 3.2. Asterisks at nodes represent posterior support values >0.97.

The phylogenetic analysis with the selected ND4 segment retrieved seven species-level lineages in *Blaesodactylus* (Figure 1). Six of these lineages correspond to the currently described species of the genus (*B. victorii*, *B. microtuberculatus*, *B. boivini*, *B. sakalava*, *B. ambonihazo* and *B. antongilensis*) (Figure 1). In addition, the available sequences of *B. sakalava* were divided into two distinct molecular clades here named *B. sakalava* clade A and *B. sakalava* clade B (Figure 1).

The phylogenetic relationships reported in our tree show that the seven species-level lineages are comprised of two different main clades: the first includes *B. victorii*, *B. microtuberculatus* and *B. boivini*, while the second comprises *B. sakalava* clade A and B, *B. ambonihazo* and *B. antongilensis* (Figure 1). In the first clade, *B. victorii* is the sister group of a clade composed of *B. microtuberculatus* and *B. boivini* (Figure 1). In the second clade, the specimens of *B. sakalava* were grouped into two different clades, together representing the sister group of the clade composed by *B. ambonihazo* and *B. antongilensis* (Figure 1).

### 3.2. Cytogenetic Analysis

**Figure 2:**
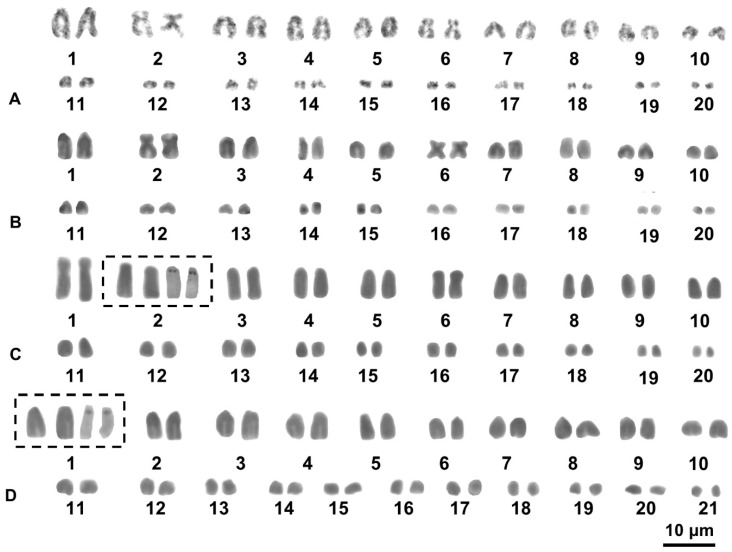
Giemsa-stained karyotypes of *B. sakalava* clade B (**A**), *B. sakalava* clade A (**B**), *B. antongilensis*, (**C**), *B. boivini* (**D**). The boxes show the NOR bearing pair, stained with Giemsa (left) and Ag-NOR staining (right).

*Blaesodactylus sakalava* and *B. antongilensis* had a karyotype of 2n = 40, mostly composed of telocentric chromosomes (Figure 2; Table 2). However, the elements of pairs 1 and 6, were metacentric in either *B. sakalava* clade A or *B. sakalava* clade B. In contrast, in *B. antongilensis*, the centromeric index (CI) of pair 1 (25.0%) corresponded to the transition value between a subtelocentric and a submetacentric element (Figure 2; Table 2) [29] .

*Blaesodactylus boivini* showed a karyotype of 2n = 42, composed of all telocentric chromosomes which gradually decrease in length (Figure 2D, Table 2).

Given the quantity and quality of metaphase plates, Ag-NOR staining and sequential C-banding + fluorochromes were successfully performed only on samples of *B*. *boivini* and *B. antongilensis*. Loci of NORs were close to the centromeres of the chromosome of pair 2 in *B. antongilensis* and to the centromeres of the elements of pair 1 in *B*. *boivini* (FGMV 3010) (Figure 2C,D).

**Figure 3:**
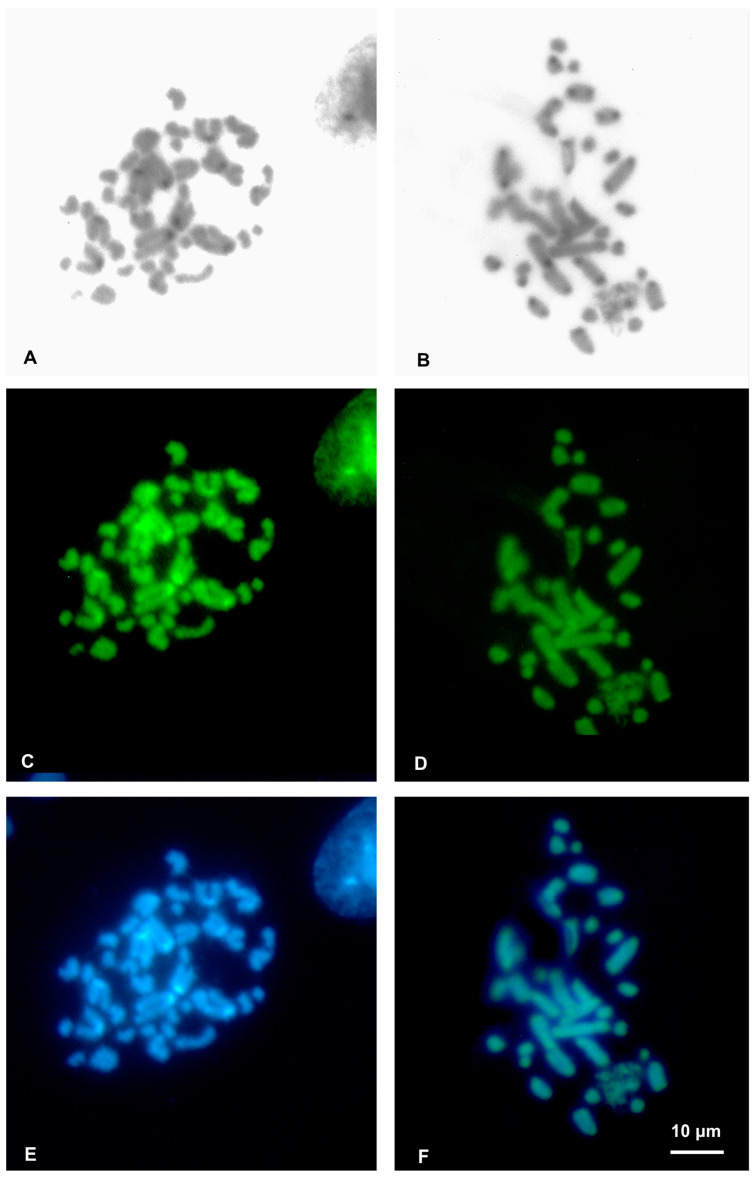
Metaphase plates of *B. boivini* (**A**,**C**,**E**) and *B. antongilensis* (**B**,**D**,**F**) sequentially stained with C-banding + Giemsa (**A**,**B**), +CMA_3_ (**C**,**D**) and +DAPI (**E**,**F**).

In both *B*. *boivini* and *B. antongilensis*, heterochromatic blocks were mostly localized on the centromeres of several chromosome pairs and were clearly evident after C-banding + Giemsa (Figure 3). In *B*. *boivini*, the main heterochromatic blocks were positive with either CMA_3_ or DAPI, while in *B. antongilensis* they were more visible with CMA_3_ (Figure 3).

## 4. Discussion

### 4.1. Molecular Analysis

The molecular and phylogenetic analysis using the selected ND4 segment allowed us to unambiguously assign all the studied samples to different *Blaesodactylus* genetic lineages as reported in Table 1 (see Figure 1). Our phylogenetic analysis using Bayesian inference showed similar evolutionary relationships and posterior support values to those previously reported by Ineich et al. [19]. All the currently recognized *Blaesodactylus* species show a relatively deep intraspecific mitochondrial genetic divergence (13–16% of ND4 uncorrected p-distance). We also highlight that the two distinct genetic clades found in *B. sakalava* (here named *B. sakalava* clade A and B) probably correspond to species-level lineages as their genetic distance is similar to that found between other *Blaesodactylus* sister species (see Figure 1) (see also [19]). This is not surprising, considering that different new *Blaesodactylus* species have been described in the last years and recent studies highlighted that the species diversity of the genus is probably still underestimated [18,19]. We also highlight that the DNA sequences of two different species of *Blaesodactylus* (*B. microtuberculatus* and *B. victorii*) are currently available from a single specimen, respectively, from the Ankarana National Park (north Madagascar) and the Tsingy limestone outcrops in the Namoroka National Park (northwestern Madagascar) (see [18,19]). In addition, most of the species-level lineages in *Blaesodactylus* appear to be allopatric, but *B. microtuberculatus* is sympatric with *B. boivini* and *B. victori* is sympatric with *B. sakalava* [18,19]. Further molecular analyses using a combination of mitochondrial and nuclear markers and a wide sampling across different Malagasy ecoregions are probably needed to better assess the molecular and species diversity in the genus *Blaesodactylus.*

### 4.2. Cytogenetic Analysis

In this study we performed the first comparative cytogenetic study on *Blaesodactylus*, providing the karyotype description of three different clades of this endemic Malagasy genus (*B. sakalava* clade A and B and *B. antongilensis*) and re-description of the karyotype of *B. boivini* (see also [20]). This contribution represents the first step in describing the karyological variability occurring in the genus *Blaesodactylus*, as well as a new contribution to reconstruct chromosomal evolutionary dynamics in a larger clade of the family Gekkonidae, which also includes the genera *Homopholis* and *Geckolepis* (Figure 1) (see also [19]).

Our chromosome analysis showed the occurrence of karyological variability between different studied species in terms of chromosome number (2n = 40–42), morphology, chromosome localization of loci of NORs, and pattern of heterochromatin. In particular, *B. sakalava* clade A and B and *B. antongilensis* showed a similar karyotype structure (with 2n = 40 and the occurrence of biarmed elements), with differences concerning the morphology of pairs 1 and 6. On the other hand, a different chromosome number (2n = 42) with only telocentric chromosomes was shown by *B. boivini*, as recently reported also by Chrostek et al. [20].

**Figure 4:**
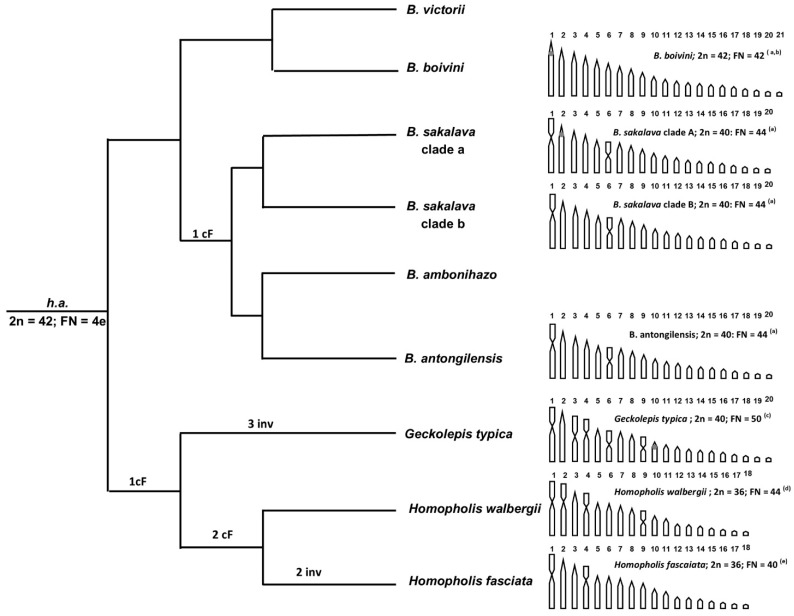
Phylogenetic relationships of *Blaesodactylus, Homopholis* and *Geckolepis* with the available haploid karyograms. h.a. = hypothesised ancestral condition, cF = centric fusion, inv = inversion. a = present paper; b = [20]; c = [7]; d = [30]; e = [31]. Phylogenetic relationships were redrawn from ([19,32], present study).

To advance a comparative hypothesis on the karyotype diversification in *Blaesodactylus* we superimposed all the currently available haploid karyograms of the genus, as well as those of the closely related genera *Homopholis* and *Geckolepis* [7,30,31], on their phylogenetic relationships ([32], this study). In particular, chromosome data are currently available for *G*. *typica* [7], *H. fasciata* and *H. walbergii* [20,30,31] (Figure 4). Notably, *G*. *typica* displays a karyotype structure similar to those of *B. sakalava* and *B. antongilensis* (2n = 40 and the occurrence of biarmed elements), while *H. fasciata* and *H. walbergii* present a lower chromosome number (2n = 36) [7,20,30,31] (Figure 4).

Among the family Gekkonidae, chromosome evolution has been hypothesized to have occurred either by an increase (as for example in the genus *Hemidactylus*) (see [33]) or by a reduction in the total chromosome number (e.g., in Diplodactylinae) [34,35].

In the case of *Blaesodactylus*, the most parsimonious chromosomal evolutionary scenario (including the lowest possible number of chromosome changes in order to generate all the different observed karyotypes) is to consider a karyotype composed of 2n = 42 and all telocentric chromosomes as the ancestral condition in the genus (Figure 4). In addition, a higher total chromosome number and a higher number of telocentric elements are generally considered ancestral karyotype characters in squamates (see e.g., [15]).

Our results show that a karyotype similar to the putative ancestral condition in *Blaesodactylus* (of 2n = 42) has probably been conserved in *B. boivini*, while different centric fusions likely occurred in *B. sakalava* clade A and B, reducing the total chromosome number to 2n = 40 and shaping the first and sixth chromosome pairs as metacentric. In *B. antongilensis*, a pericentromeric inversion (or, alternatively, a centromere repositioning) [36] likely shaped the first chromosome pair as a submeta-/subtelocentric chromosome (Figure 4). Similarly, in the clade including *Geckolepis* and *Homopholis* two centric fusions probably occurred in their common ancestor, producing a reduction in the total chromosome number (from 2n = 42 to 2n = 40) and the formation of two metacentric pairs (Figure 4). *Geckolepis typica* conserved a karyotype composed of 2n = 40 chromosomes, but three inversions produced an increase in biarmed elements (pairs 3, 5 and 8) (Figure 4). Two additional centric fusions occurred in *Homopholis* reducing the chromosome number to 2n = 36 and producing two additional biarmed chromosome pairs (pairs 2 and 8) (2n = 36; FN = 44). During the chromosomal diversification of this genus, *H. walbergii* conserved the primitive *Homopholis* karyotype, while two inversions occurred in *H. fasciata* (2n = 36; FN = 40) (Figure 4). Other than the hypothesized chromosome inversion, it is also possible that centromere repositioning has been involved in generating the chromosomal variability currently observable in *Geckolepis* and *Homopholis*.

Similar examples of a reduction in the total chromosome number and of a progressive formation of biarmed elements have been observed in different gecko genera, including *Uroplatus*, *Lygodactylus*, *Matoatoa*, *Paroedura* and *Christinus* [9,37,38,39,40]. In particular, all these different gecko genera show a karyotype composed of 2n = 34–42 mostly telocentric chromosomes and are characterized by the progressive appearance of biarmed elements by means of chromosome fusions in karyotypes with a reduced chromosome number and/or the translocation of small NOR-bearing chromosomes on larger chromosomes.

The repeated observation in different gecko groups of similar, independent instances of reduction in the total chromosome number by means of chromosome fusions and inversions suggests the possible occurrence of a convergent karyotype evolution.

Concerning the chromosome localization of NORs, they are close to the centromeres of the chromosomes of the first pair in *B*. *boivini* (see also [20]). Probably, this configuration is also conserved in *B. sakalava* clade A. In fact, this clade shows NORs on the second (telocentric) pair, but its first (metacentric) pair likely derived from the centric fusion of smaller telocentric chromosomes. In *G*. *typica*, NORs are close to the centromeres of the chromosomes of pair 10, but considering the lack of information on *Homopholis*, as well as on other *Geckolepis* species, additional data are needed to advance any hypothesis on NOR diversification.

Analysis with C-banding did not reveal the presence of differentiated sex chromosomes in either *B. boivini* or *B. sakalava* clade A. Nevertheless, we cannot exclude the possible presence of mostly pseudoautosomal sex chromosomes at an early stage of differentiation, which are known to occur in different groups of geckos (e.g., [9,15,39,41,42]). Alternatively, it is also possible that the sex is determined by environmental factors in the genus *Blaesodactylus* (e.g., temperature-dependent sex determination) as it has been previously documented in several gecko species (see [15]). Future research should employ a combination of molecular and cytogenetic methods to uncover the mechanism of sex determination in this group of geckos. Overall, C-banding + Giemsa produced similar results in *B. boivini* and *B. antongilensis*, evidencing a relatively low content of heterochromatin which is mostly localized at the centromeric regions of several chromosome pairs and particularly evident on the largest pairs (see Figure 3). This result was expected considering that a low content of heterochromatin generally characterizes the genome of squamate reptiles and is asymmetrically abundant on fully differentiated sex chromosome pairs (e.g., [15,43]). The differences between *B. boivini* and *B. antontongilensis* concerning the results obtained with C-banding + CMA_3_ and C-banding + DAPI (which highlight CG- and AT-rich regions, respectively) suggest that a different nucleotide composition might characterize the repeated DNA content of the two species.

## 5. Conclusions

We performed a molecular analysis and a comparative cytogenetic study on four taxa of *Blaesodactylus*, presenting the first karyotype description of three of them. We show the occurrence of karyological variability in the genus concerning the total chromosome number (2n = 40–42), chromosome morphology, the karyotype localization of loci of NORs, and pattern of heterochromatin.

Our findings allowed us to hypothesize that the chromosomal diversification in the genus involved a reduction in the chromosome number and the progressive formation of biarmed chromosomes by means of fusions and inversions (or centromere repositioning). Following this hypothesis, the ancestral karyotype condition in *Blaesodactylus* is 2n = 42, similar to that shown by *B. boivini*, while apomorphic conditions of 2n = 40 with the formation of different biarmed chromosome pairs were observed in *B. sakalava* and *B. antongilensis*. Comparing our data to those available from the literature, we suggest that comparable chromosome rearrangements might also have characterized the karyotype evolution of the closely related *Geckolepis* and *Homopholis*. We also highlight that a similar pattern of chromosome diversification has already been observed in other gecko genera (e.g., *Paroedura*, *Lygodactylus*, *Matoatoa*, and *Uroplatus*), possibly representing independent events of convergent karyotype evolution.

This contribution confirms that chromosome changes often characterize the evolution and diversification of independent lineages of Malagasy squamates and that comparative cytogenetics provides a useful set of tools to describe evolutionary dynamics.

## Figures and Tables

**Table 1:** Taxonomic attribution, origin, sex, field number of the study specimens of *Blaesodactylus*.

Species	Specimen	Locality	Sex
*B. antongilensis*	GA 483	Masobe Forest, Betampona	female
*B. antongilensis*	GA 1049	Masobe Forest, Betampona	female
*B. boivini*	FGMV 3010	Montagne des Français	female
*B. sakalava* clade A	FGMV 2029	Ifaty	female
*B. sakalava* clade A	FGMV 2030	Ifaty	male
*B. sakalava* clade A	FGMV 2032	Ifaty	male
*B. sakalava* clade B	GA 80	Isalo	male
*B. sakalava* clade B	GA 376	Marofandilia	juvenile
*B. sakalava* clade B	GA 377	Marofandilia	male
*B. sakalava* clade B	GA 378	Marofandilia	male

**Table 2:** Chromosome morphometric parameters of the study species. RL% = Relative Length (length a chromosome/total chromosome length × 100). m = metacentric; sm = submetacentric; t = telocentric.

Species	*B. antongilensis*	*B. boivini*	*B. sakalava* cl. A	*B. sakalava* cl.B
Chrom.	RL%	RL%	RL%	RL%
1	9.6 ± 0.7 25.0 ± 4.0 (st/sm)	9.7 ± 0.6 (t)	9.8 ± 1.0 39.3 ± 4.5 (m)	9.1 ± 0.8 43.1 ± 3.4 (m)
2	8.7 ± 1.1 (t)	8.2 ± 0.4 (t)	8.2 ± 0.9 (t)	8.1 ± 0.9 (t)
3	7.4 ± 0.4 (t)	7.4 ± 1.2 (t)	8.1 ± 0.7 (t)	7.8 ± 0.6 (t)
4	7.3 ± 0.5 (t)	6.6 ± 0.9 (t)	7.1 ± 0.6 (t)	6.6± 0.8 (t)
5	7.0 ± 0.7 (t)	5.8 ± 0.7 (t)	6.8.1 ± 0.5 (t)	6.4 ± 0.6 (t)
6	6.7 ± 0.6 38.4 ± 3.5 (m)	5.8 ± 1.0 (t)	6.2 ± 0.8 47.0 ± 3.0 (m)	6.2 ± 0.6 46.3 ± 3.1 (m)
7	6.2 ± 0.9 (t)	5.7 ± 0.5 (t)	5.4 ± 0.6 (t)	6.1 ± 0.8 (t)
8	5.6 ± 0.8 (t)	5.6 ± 0.4 (t)	5.3 ± 0.7 (t)	5.7 ± 0.6 (t)
9	5.0 ± 0.6 (t)	5.6 ± 0.6 (t)	5.3 ±0.8 (t)	5.4 ± 0.5 (t)
10	4.9 ± 0.5 (t)	5.6 ± 0.6 (t)	5.2 ± 0,6 (t)	4.9 ± 0.4 (t)
11	4.8 ± 0.8 (t)	4.6 ± 0.5 (t)	4.2 ± 0.6 (t)	4.5 ± 0.6 (t)
12	4.6 ± 0.4 (t)	3.9 ± 0.5 (t)	4.0 ± 0.4 (t)	4.1 ± 0.7 (t)
13	4.0 ± 0.6 (t)	3.3 ± 0.4 (t)	3.8 ± 0.5 (t)	3.8 ± 0.7 (t)
14	3.3 ± 1.0 (t)	3.3 ± 0.8 (t)	3.7 ± 0.7 (t)	3.6 ± 0.9 (t)
15	3.2 ± 0.8 (t)	3.2 ± 0.6 (t)	3.7 ± 0.7 (t)	3.4 ± 0.5 (t)
16	2.8 ± 0.9 (t)	3.2 ± 0.7 (t)	3.5 ± 0.6 (t)	3.1 ± 0.8 (t)
17	2.6 ± 0.7 (t)	2.9 ± 0.5 (t)	3.3 ± 0.6 (t)	3.0 ± 0.5 (t)
18	2.3 ± 0.6 (t)	2.8 ± 0.4 (t)	2.8 ± 0.7 (t)	2.8 ± 0.4 (t)
19	2.0 ± 0.5 (t)	2.4 ± 0.4 (t)	2.7 ± 0.8 (t)	2.7 ± 0.6 (t)
20	2.0 ± 0.8 (t)	2.3 ± 0.3 (t)	2.6 ± 0.5 (t)	2.6 ± 0.7 (t)
21		1.9 ± 0.5 (t)		

## Data Availability

Newly generated cytogenetic data are available within this manuscript. DNA sequences are available on GenBank (accession numbers: OR113357-OR113363).
